# Fluid Dynamics of Coarctation of the Aorta and Effect of Bicuspid Aortic Valve

**DOI:** 10.1371/journal.pone.0072394

**Published:** 2013-08-27

**Authors:** Zahra Keshavarz-Motamed, Julio Garcia, Lyes Kadem

**Affiliations:** 1 Laboratory of Biorheology and Medical Ultrasonics, University of Montreal Hospital Research Center (CRCHUM), Montréal, Québec, Canada; 2 Biomedical Engineering Department, University of Montreal, Montréal, Québec, Canada; 3 Laboratory of Cardiovascular Fluid Dynamics, Mechanical and Industrial Engineering Department, Concordia University, Montréal, Québec, Canada; 4 Québec Heart and Lung Institute, Laval University, Québec, Québec, Canada; 5 Department of Radiology, Northwestern University, Chicago, Illinois, United States of America; University of California, San Diego, United States of America

## Abstract

Up to 80% of patients with coarctation of the aorta (COA) have a bicuspid aortic valve (BAV). Patients with COA and BAV have elevated risks of aortic complications despite successful surgical repair. The development of such complications involves the interplay between the mechanical forces applied on the artery and the biological processes occurring at the cellular level. The focus of this study is on hemodynamic modifications induced in the aorta in the presence of a COA and a BAV. For this purpose, numerical investigations and magnetic resonance imaging measurements were conducted with different configurations: (1) normal: normal aorta and normal aortic valve; (2) isolated COA: aorta with COA (75% reduction by area) and normal aortic valve; (3) complex COA: aorta with the same severity of COA (75% reduction by area) and BAV. The results show that the coexistence of COA and BAV significantly alters blood flow in the aorta with a significant increase in the maximal velocity, secondary flow, pressure loss, time-averaged wall shear stress and oscillatory shear index downstream of the COA. These findings can contribute to a better understanding of why patients with complex COA have adverse outcome even following a successful surgery.

## Introduction

Coarctation of the aorta (COA) is a congenital heart disease characterized by narrowing in the isthmus zone, i.e., the section of the descending aorta distal to the left subclavian artery. It accounts for 5–10% of congenital heart diseases and represents 7% of all critically ill infants with heart disease [1]. Up to 85% of patients with COA have a bicuspid aortic valve (BAV) [2], [3], [4]. Oliver and colleagues (2004) reported that the prevalence of aortic complications in patients with COA was 22% if a BAV was present compared with 8% in patients without BAV [5]. The most frequent complications are ascending and descending aortic aneurysms. Other complications may also exist such as false aneurysm, aortic dissection and aortic rupture [5], [6], [7], [8]. When COA coexists with BAV, the burden on the left ventricle significantly increases which can lead to heart failure [2], [9]. Additionally, patients with COA and BAV are at higher risk of developing secondary complications even after successful surgical repair both directly related to intervention and secondary to systemic arteriopathy. Indeed, in the cohort of patients with COA investigated by Oliver et al. (2004), from the 10 cases of aneurysm, developed at the site of the previous COA repair, 8 patients had both COA and BAV. As a consequence, despite advancements in surgical techniques, life expectancy for patients with COA and BAV remains reduced due to long-term morbidity [5].

It has been hypothesized that these sources of morbidity can be explained on the basis of adverse hemodynamic and vascular biomechanics leading to the progression of cardiovascular diseases [10], [11], [12]. Adverse hemodynamic conditions are often categorized by disturbed and turbulent flow, leading to abnormal flow patterns and elevated wall shear stresses that can result in the degeneration of the arterial wall vessel, atherosclerosis, and aneurysm initiation [13]. Beyond this, since disturbed flow strongly influences vascular pathogenesis, and vice versa, flow information can be greatly useful for diagnostic purposes. Proper characterization of flow in large vessels has strong potential to help for treatment planning. Particularly, since most cardiovascular interventions intend to restore normal, or improved flow in cases of the disease, detailed information of pre-operative flow conditions, or the capability to predict postoperative flow conditions resulting from a specific intervention, can have impressive clinical impact [14].

The aim of the present work is, therefore, to investigate how the presence of a COA and a BAV affects the flow field characteristics and, in particular, wall shear stress in the aorta. The emphasis is on the effect of the BAV on the COA fluid dynamics using numerical simulations and magnetic resonance imaging (MRI) measurements. This is the first step towards establishing guidelines for the treatment of patients with COA and BAV based on pre-existing hemodynamics conditions (i.e., some surgical techniques may not be optimal for patients with both COA and BAV, then, more aggressive surgical approaches may be required). For this purpose, numerical simulations and MRI measurements were performed in a complete realistic three-dimensional model of the aorta (including: ascending aorta, aortic branches and descending aorta) with two different models of the aorta: normal aorta and COA (75% reduction by area) in the presence of two different aortic valve conditions: normal and bicuspid.

## Methods

### Numerical Simulations

#### Numerical model

**Figure 1:**
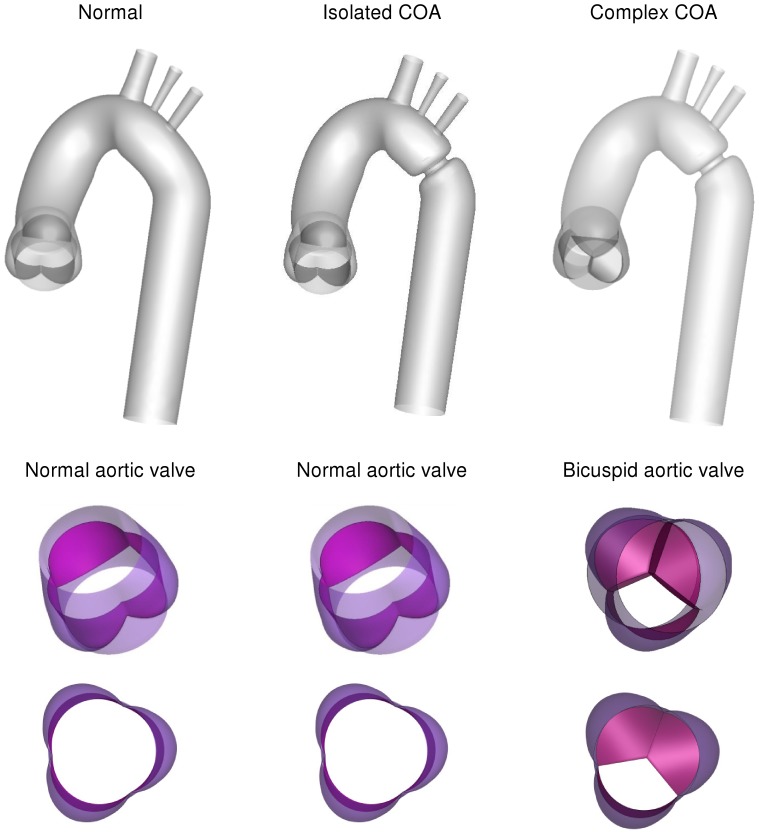
Three different geometries considered for the numerical simulations. a normal tricuspid aortic valve and a normal aorta representing normal case, a normal aortic tricuspid valve and an aorta with COA (75% by area), representing isolated COA, a bicuspid aortic valve and an aorta with COA (75% by area), representing complex COA.

Numerical simulations were carried out for three different cases: (1) a normal tricuspid aortic valve with effective orifice area (EOA) of 3 cm^2^ was placed at the inlet of the anatomical model of the normal aorta, representing normal case, (2) a normal aortic tricuspid valve (EOA = 3 cm^2^) was placed at the inlet of the aorta with COA (75% by area), representing isolated COA, (3) a bicuspid aortic valve (EOA = 1.1 cm^2^) was placed at the inlet of the aorta with COA (75% by area), representing complex COA (Fig.1). The aorta model was created based on clinical MRI images obtained in an adult patient. Realistic aortic valve models were added to the aorta model [15].

Computations were performed using computational fluid dynamics open source (OpenFOAM) based on finite volume method. In healthy vessels, the blood flow is usually laminar and does not experience transition to turbulence. The solution was therefore obtained by simulating a laminar flow inside the domain of healthy aorta. Under physiological conditions, the blood flow may remain laminar proximal (upstream) to moderate and severe stenoses but becomes transitional or turbulent distally [16], [17], [18]. As physiological flows are exclusively in the low-Re range, the 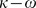 model [19], which is primarily intended for simulating low-Re internal flows (Re <10,000) was used. Therefore, models with COA and COA+BAV were investigated using the transitional 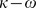 turbulence model, which has been shown to give a good overall representation of both steady and pulsatile flow [20], [21].

Mesh independency in the study was judged by two criteria: velocity and wall shear stress. Mesh definition was considered as acceptable when no significant difference (lower than 5%) between successive meshes was noticed in the wall shear stress along the wall of the aorta, and also in velocity profiles at different locations of the aorta specifically downstream of the aortic valves and COA. Mesh independency was achieved for three cases with 900000 elements (normal case), 8713200 elements (isolated COA) and 10500000 (complex COA). The mesh is of hybrid character and consists of hexahedral elements. Complex geometrical regions are discretized with unstructured tetrahedral and wedge elements (i.e., the aortic root, the valve housing, the leaflets and aortic arch branches). The grid is clustered near the COA. Moreover, additional care was taken near the wall to maintain y^+^ less than 1 as criterion required by 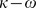 model. For time independency, several time steps were tested: 0.001 s, 0.002 s and 0.0025 s and 0.003 s. The solution marched in time with a time step 0.0025 s to satisfy time independency. Four cardiac cycles were simulated for each flow model to ensure periodicity. The convergence was obtained when all residuals reached a value lower than 10^−5^.

The total shear stress exerted on the wall throughout the cardiac cycle was evaluated using the time-averaged wall shear stress (TAWSS) which is obtained as follow:
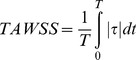
(1)


Here, T and 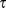
are the cardiac cycle period and instantaneous wall shear stress, respectively.

To evaluate temporal oscillations in wall shear stress, the oscillatory shear index (OSI) was used as follow:
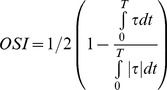
(2)


Additionally, CFD uncertainty and error in the study were analyzed [22]. Table 1 shows the calculations for the discretization error for wall shear stress. The parameters, 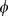, 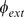, 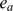, 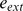 and 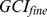 represent the wall shear stress, the extrapolated wall shear stress value, the approximate relative error, the extrapolated relative error and the fine-grid convergence index, respectively [22]. It should be mentioned for this analysis, the wall shear stress (Pa) was determined at the neck of the COA (inner wall). These computations indicate that the numerical uncertainty is 1.07% for complex COA (Table 1), 0.275% for isolated COA and 3.718% for normal case as well.

**Table 1:** Calculation of discretization errors for three cases simulated in this study.

	Normal	Isolated COA	Complex COA
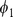 (Pa)	3.35	78.88	111
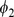 (Pa)	3.31	78.81	108.5
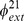 (Pa)	3.37	79.1	112
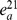	1.19%	0.088%	2.25%
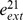	0.59%	0.278%	0.9%
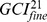	3.718%	0.275%	1.07%

The calculations were performed for the wall shear stress at the neck of the complex COA (inner wall). 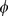, 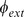, 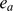, 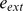 and 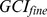 represent the wall shear stress, the extrapolated wall shear stress value, the approximate relative error, the extrapolated relative error and the fine-grid convergence index, respectively.

#### Boundary conditions and model properties

Blood was assumed to be a Newtonian and incompressible fluid with dynamic viscosity of 0.0035 Pa·s and a density of 1050 kg/m^3^ [23]. Although whole human blood tends to exhibit non-Newtonian behavior at shear rates under 100 s^−1 ^near the vessel walls, the shear rates in large arteries are generally observed to be greater than 100 s^−1^ and therefore it is reasonable to assume a Newtonian fluid in the simulation [24]. The arterial wall was treated as solid and rigid. This can be justified by: 1) Jin et al. (2003) showed that rigid wall assumption for the aorta is realistic. Their results showed that the overall behavior for wall shear stress at each point is similar for the rigid and elastic walls with average root mean squared error of 1.23% [25]. Furthermore, their velocity distribution, computed in both elastic and rigid models, showed good agreement with MRI velocity measurements; 2) it was reported that patients with COA are usually hypertensive and characterized by reduced compliance and elevated stiffness index in both proximal and distal aorta [26], [27], [28], [29], [30]. 3) In this study, the good agreement between numerical simulations, including rigid wall, and MRI velocity measurements, which includes elastic aorta, justified rigid wall as a quite well assumption. Non-permeable and a no-slip boundary condition was applied at the rigid walls.

Under normal conditions (no COA) a small portion of the total flow rate (15%) is directed towards aortic arch branches (divided equally between three branches) and the rest (85% of total inlet flow rate) through COA and the descending aorta. However, when a COA is present, depending on its severity, a larger portion of the total flow rate bypasses the COA [9]. In this study, following predictions from the lumped parameter model with respect to the severity of the COA (75% COA), 30% of total inlet flow rate was specified at the exit of the branches (divided equally between three branches) and the rest (70% of total inlet flow arte) through COA and the descending aorta [9]. Maintaining the same flow fraction between aorta branches helped us to simplify the problem by assuming that the possible small differences between flow rates of branches have insignificant effects on the flow field in the aorta especially in the COA region. This approach minimizes the number of parameters to be measured and it is suitable for clinical settings since all parameters can be measured non-invasively [9]. Indeed, measurement of flow rate through each branch is not possible in Doppler echocardiography and requires measurement on several planes in magnetic resonance imaging which significantly increases the acquisition time. An alternative approach will be to couple each outlet of the numerical model to a lumped-parameter model. Kim et al. (2009) adopted this approach in their model [31]. Although this approach may also lead to correct pressure field but it requires input parameters that cannot be measured *in vivo*.

**Figure 2:**
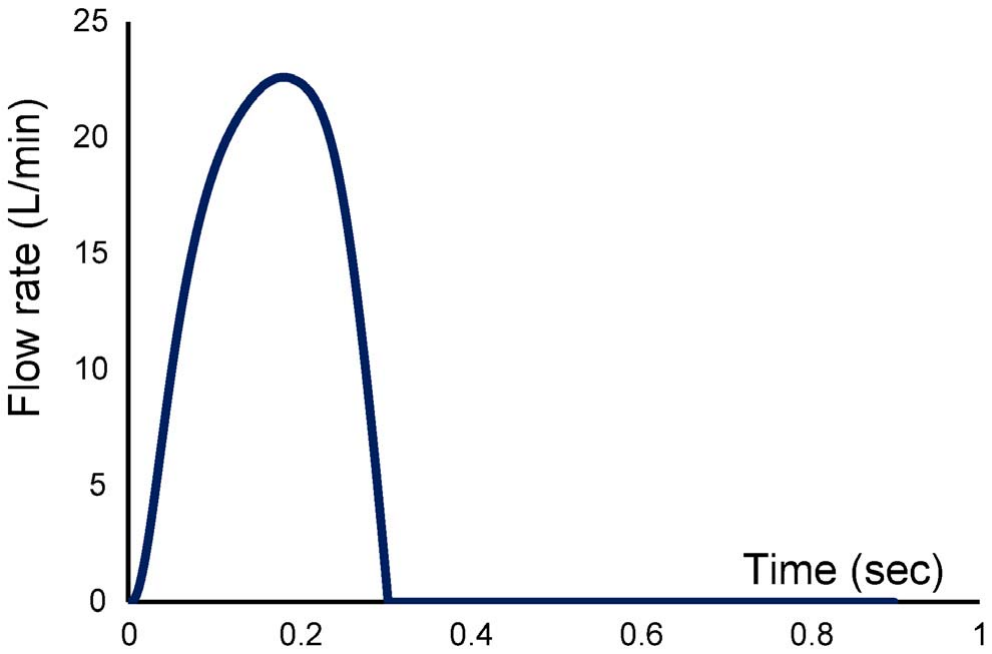
Pulsatile flow rate waveform used as inlet condition for the numerical simulations.

In order to start the pulsatile cycle calculations, a steady state solution at the peak of the systole was first obtained. This steady state solution was then used as the initial condition for the unsteady computations. For pulsatile simulations, a flat velocity profile with a pulsatile waveform (Fig. 2) was applied upstream of the aortic valve (tricuspid and bicuspid), in the left ventricle outflow tract [32]. Applying a flat velocity profile at the described location is justified by *in vivo* hot film anemometry measurements in various animal models [33], [34]. All numerical simulations were performed under a pulsatile mean flow rate of 5 L/min with a systolic duration of 300 ms and a heart rate of 70 bpm. The conditions of the simulations have been chosen so that the non-dimensional parameters are within the physiological range. This corresponded to mean systolic inlet Reynolds numbers of 2400, Dean number of 1231 and a Womersley number of 16.5.

### Magnetic Resonance Imaging (MRI)

**Figure 3:**
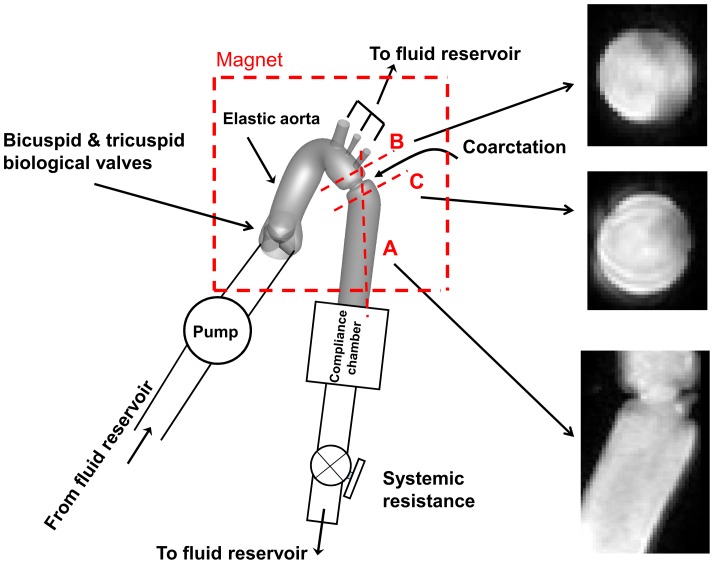
Schematic diagram of the *in vitro* flow model used for MRI measurements. Dashed red lines show the planes measured with MRI.

For the purpose of this study, we used our previously described and validated *in vitro* model [9], [35]. It is composed of a fluid reservoir, a gear pump, elastic models of the aorta and adjustable systemic arterial resistance and compliance (Fig. 3). A mixture of 65% saline and 35% glycerine, in volume at room temperature, is used to mimic viscous proprieties of blood at 37°C [36]. The fluid is pumped from a reservoir, crosses the aortic valve and then is directed towards the aortic branches and the descending aorta. In this study, flow through aortic branches was adjusted with respect to the severity of COA following the predictions from our recent lumped parameter model of flow through a COA (see Keshavarz-Motamed et al., 2011 for more details) [9]. The flow proportions (upper-body *vs* lower-body) used for MRI measurements are identical to those used in the numerical simulations (Isolated COA and complex COA: 70% of total inlet flow rate goes through the COA and the rest through aortic arch branches; normal: 85% of total inlet flow rate goes through descending aorta and the rest through aortic arch branches). Including aortic arch branches is then essential for the investigation of COA hemodynamics in order to avoid flow overestimation through the COA. Instantaneous flow rates were measured by T206 Transonic flowmeter (Transonic System Inc., Ithaca, NY, USA, accuracy of 1% full scale) at the level of descending aorta and aortic arch arteries. The pressures upstream (10 mm) and downstream (10 mm) of the aortic valve were measured using Truwave disposable pressure transducers (Edwards Lifesciences, Irvine, California, USA, sensitivity of 5 µV/V/mmHg±1%) in order to measure left ventricle and aorta pressures during MRI scanning. The geometries of the aorta and the aortic valve used for MRI measurements are identical to those used in numerical simulations (Fig. 1). Aortic valves used for MRI measurements are trileaflet biological normal (Mitroflow, EOA = 3 cm^2^) and bicuspid (Mitroflow, EOA = 1.1 cm^2^) with a mean flow rate of 5 L/min (Fig. 2). In this study, the type of the bicuspid aortic valve (BAV) formed by the fusion of two normal cusps of the normal trileaflet aortic valve, leading to a moderate-to-severe narrowing at the valve level, was considered. Such BAV configuration with a single raphe is the most frequent (88% of cases) [37]. The elastic model of the aorta used here has a radial dilation of the proximal aorta of 8% (physiological value around 10%) [38] and a total arterial compliance of 1.75 ml/mmHg (physiological value 1.84±0.76 ml/mmHg [39].

The aortic model was placed at the center of the magnet during the tests and all data were collected with the use of a clinical 3 Tesla magnetic resonance scanner with a dedicated phase-array receiver coil (Achieva, Philips Medical Systems, Best, Netherlands). An ECG patient simulator (model 214B, DNI Nevada Inc, USA) was used to synchronize scanner gating with the PC controllable pump. A standard examination was performed by initial acquisition of SSFP cine images in standard longitudinal and transverse planes for acquisition planning.

Phase-contrast (sQFlow Phase SENSE) retrospective examination was performed on three planes: transverse planes (10 mm) downstream and upstream of the COA and a plane perpendicular to the COA (Fig. 3). MRI imaging parameters consisted of: TR/TE (17.99/3.97 ms), flip angle (15°), pixel spacing (1.66 mm), slice thickness (10 mm), acquisition matrix (256 × 256) and encoding velocity (2 × maximal velocity).

A custom-made research application was developed using Matlab software (Mathworks, Natick, Ma) to process and analyze MRI images [40]. Spatial resolution of MRI images (initial resolution: 1.66 mm) was artificially improved by a factor of three using a bicubic averaged interpolation (final resolution: 0.42 mm) and magnitude image stack was processed to filter background noise. Other methods have been used to calculate the flow from MRI images (e.g., Wong et al., 2010) [41].

## Results

### Comparison of Numerical Simulation *vs.* MRI Measurements

**Figure 4:**
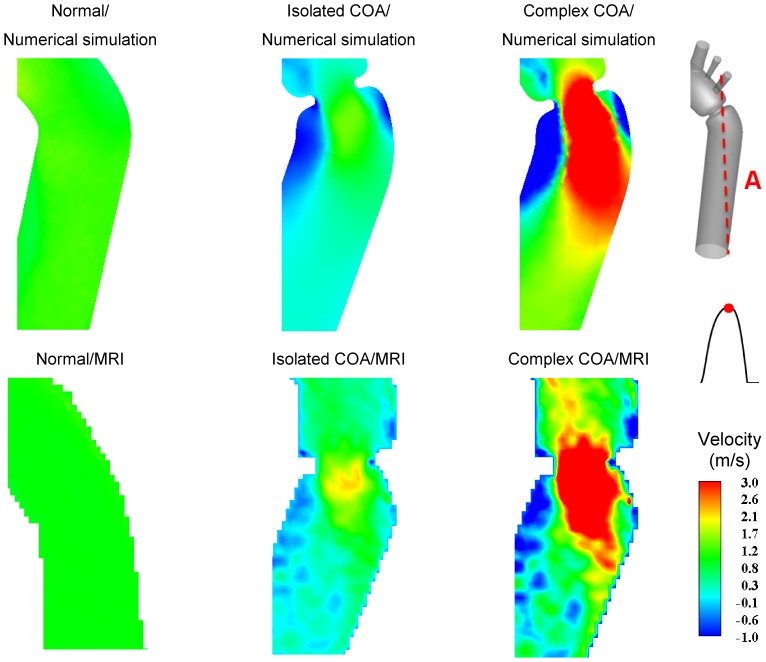
Axial velocity contours at cross section A (the longitudinal cross-section passing through the COA). Results obtained numerically and measured with MRI at peak of the systole.

**Figure 5:**
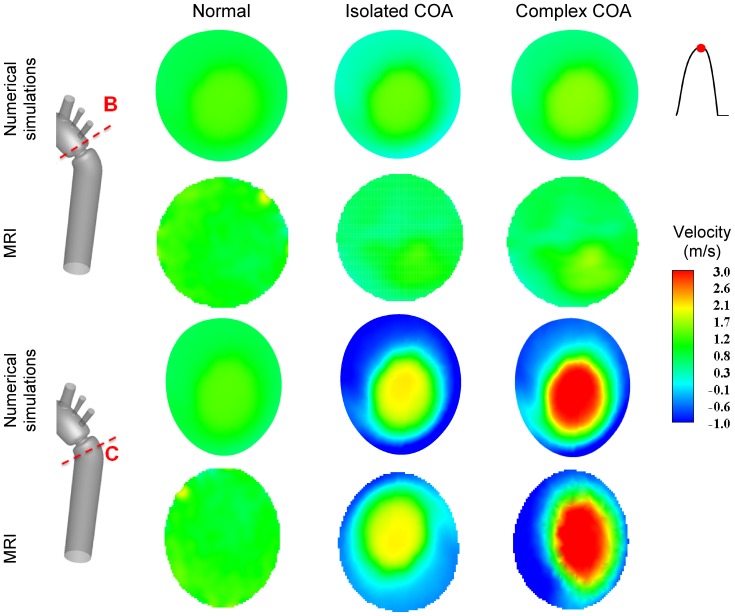
Axial velocity contours at cross sections B (upstream from COA) and C (downstream of COA). Results obtained numerically and measured with MRI at peak of the systole.

**Figure 6:**
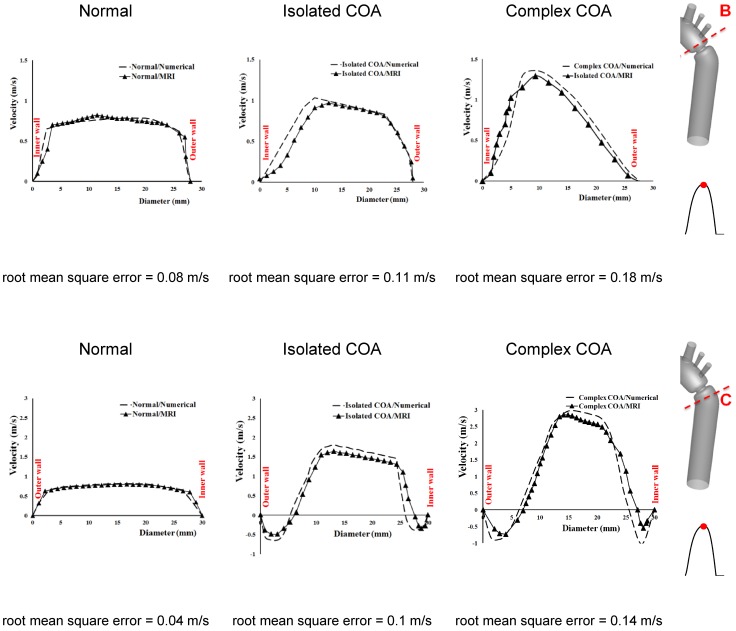
Velocity profiles along diameter upstream (section B) and downstream of the COA (section C) at peak of the systole.

**Figure 7:**
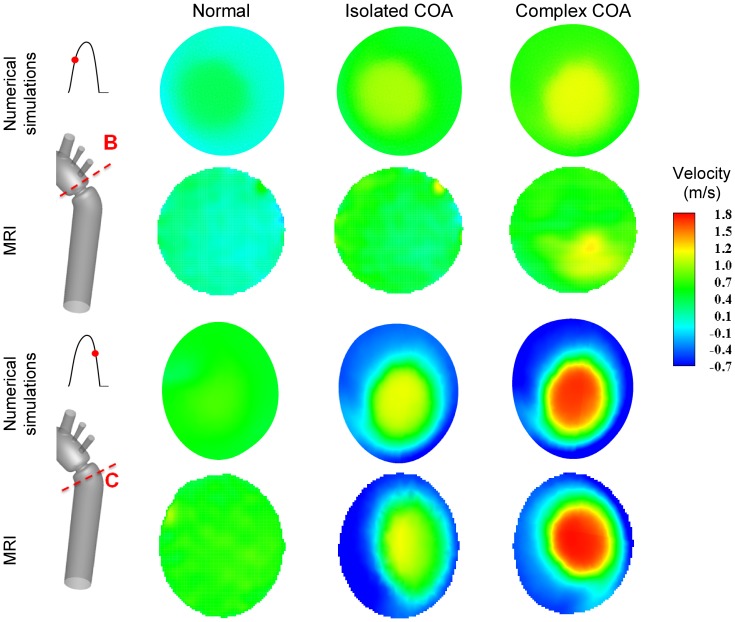
Axial velocity contours at cross section B (upstream from COA) during acceleration and cross section C (downstream of COA) during deceleration. Results obtained numerically and measured with MRI.

**Figure 8:**
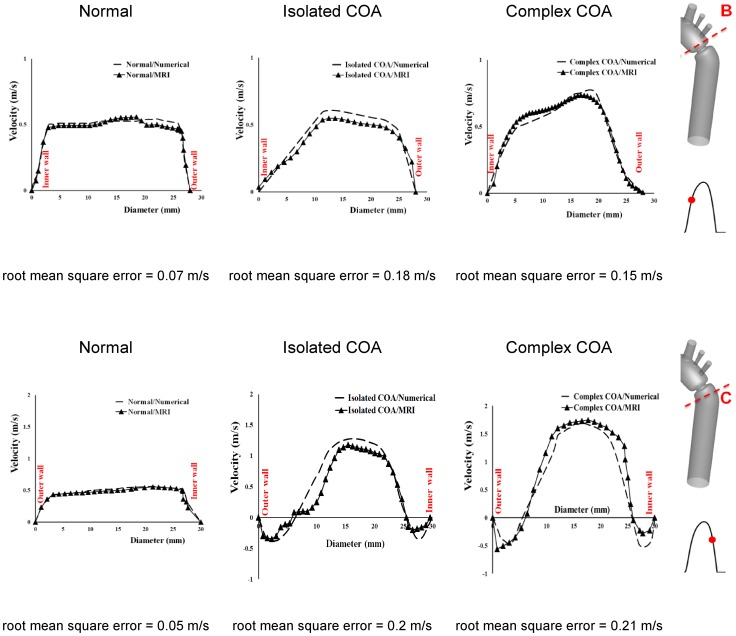
Velocity profiles along diameter at cross section B (upstream from COA) during acceleration and cross section C (downstream of COA) during deceleration.

Figure 4 shows velocity contours on the longitudinal cross-section (A) passing through the COA obtained numerically and measured with MRI for all three cases investigated in this study: normal, isolated COA and complex COA. Figure 5 represents the axial velocity contours obtained both numerically and experimentally on a cross section upstream and downstream of the COA (sections B and C) at the peak of the systole. The results show very good qualitative agreements between numerical simulations and MRI in all cases. More quantitatively, Figure 6 displays the velocity profiles along a diameter upstream and downstream of the COA for all three cases at the peak of the systole. There is good quantitative agreements between the velocity profiles obtained numerically and experimentally with root mean square errors between 0.04 and 0.18 m/s. Figure 7 shows good qualitative agreements between the numerical and experimental axial velocity contours on the cross section upstream of the COA (section B) at an instant during acceleration and on the cross section downstream of the COA (section C) at an instant during deceleration. Figure 8 displays the velocity profiles along a diameter upstream from the COA at an instant during acceleration and downstream of the COA at an instant during deceleration for all three cases investigated in this study. Root mean square errors between numerical and experimental velocity profiles are between 0.05 and 0.21 m/s. The good agreements between measured and computed velocity profiles permit us to interrogate numerical solutions with confidence to elucidate flow features that are hard to access through MRI measurements (e.g., time-averaged wall shear stress).

### Flow Dynamics of a Normal Aorta and Aorta with COA

**Figure 9:**
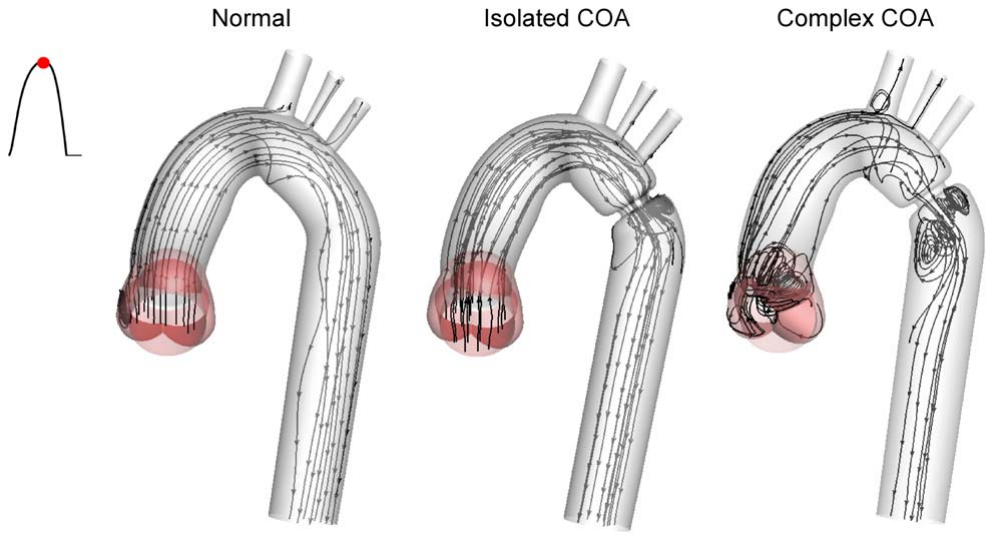
Instantaneous streamlines at peak of the systole. In the case of a normal aorta, the flow is laminar and fully attached to the wall. The presence of a COA significantly alters the flow dynamics in the aorta. The flow detaches from the aortic wall and develops into a high-speed eccentric jet downstream of the COA and the BAV.

In the case of a normal aorta, the flow is laminar and fully attached to the wall. The velocity profile is uniform with a relatively low magnitude of 0.8 m/s (Figures 4, 5 and 7). No vortex can be observed through the whole aorta at the peak of the systole (Fig. 9).

The presence of a COA alters significantly the flow dynamics in the aorta. As the flow exits the COA, the fluid cannot abruptly change direction and follow the steep curvature to reattach to the descending aorta wall (Fig. 4). The disturbed flow resulting from COA detaches from the walls and develops into a high-speed eccentric jet with maximal velocities of: 1.9 m/s for isolated COA and 3 m/s for complex COA. The eccentricity of the jet is mainly due to the curvature of the aorta (R = 3.6 cm) since the COA is almost symmetric. Under these conditions, the maximum axial velocity no longer occurs at the centerline but a skewed velocity profile develops instead where higher velocities occur near the outer wall, as already reported for a curved tube with simplified models of COA and BAV [32]. The high speed jet induces strong reversed flow and recirculation areas along both the inner and outer walls (Figures 4, 5 and 7). Strong vortices are generated with elevated negative velocities: up to 0.5 m/s for isolated COA and up to −1 m/s for complex COA. The reversed flow and recirculation areas described are further demonstrated by plotting the instantaneous velocity streamlines in the entire computational domain for the three different configurations simulated in this study (Fig. 9). Significant recirculation zones appear in the COA region which are clearly shown by the instantaneous streamlines plotted. In the case of complex COA, the sizes of the vortices and recirculation areas created in the flow field are enlarged more comparing to those in isolated COA. This demonstrates the effect of the presence of the BAV on the flow dynamics downstream of a COA. The cyclic motion of vortices and the reverse flow at the COA region (in both isolated and complex COA cases) have opportunities to cause atherosclerosis, as pointed out by Yearwood and Chandran (1980, 1984) [42], [43].

**Figure 10:**
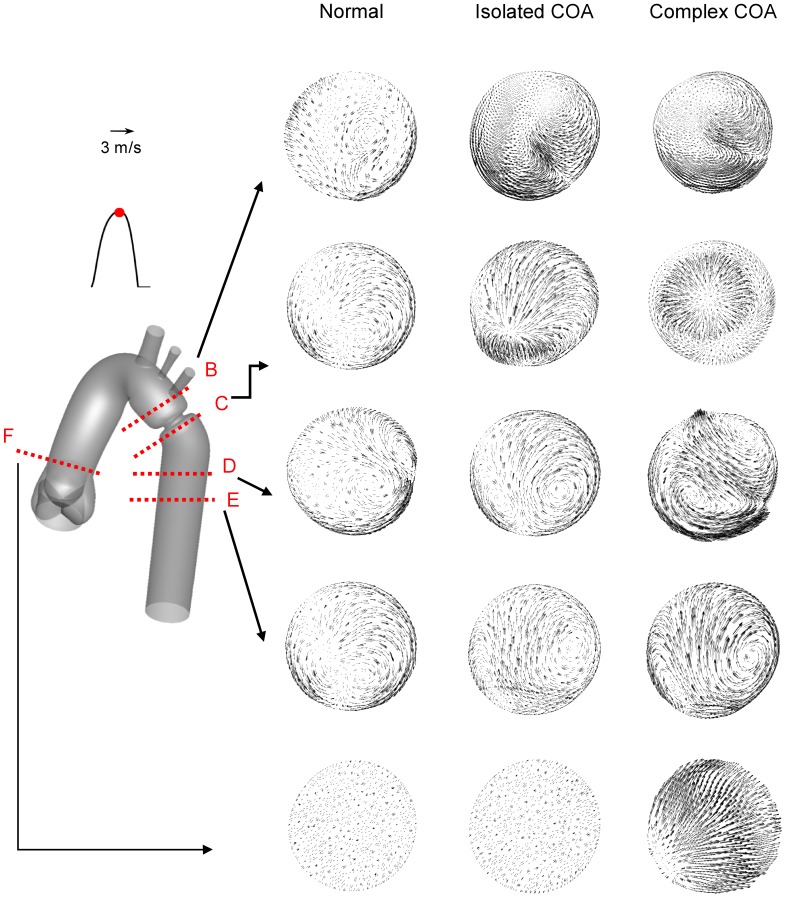
Secondary flow obtained numerically at different cross-sections at the peak of the systole. The presence of both BAV and COA significantly intensifies the amplitude of the secondary flow mainly downstream of COA.

Figure 10 shows the secondary flow for normal, isolated COA and complex COA cases. For normal case, as a result of elevated axial velocity and centrifugal force, two weak contra-rotating vortices exist. In the case of isolated COA, the vortices grow stronger and larger and cover much of the cross-section. Furthermore, if the COA is associated with the BAV, the secondary flows are more significant mainly downstream of the COA. This intensification of the secondary flow pattern will significantly contribute to modification in TAWSS distribution on the aortic wall.

**Figure 11:**
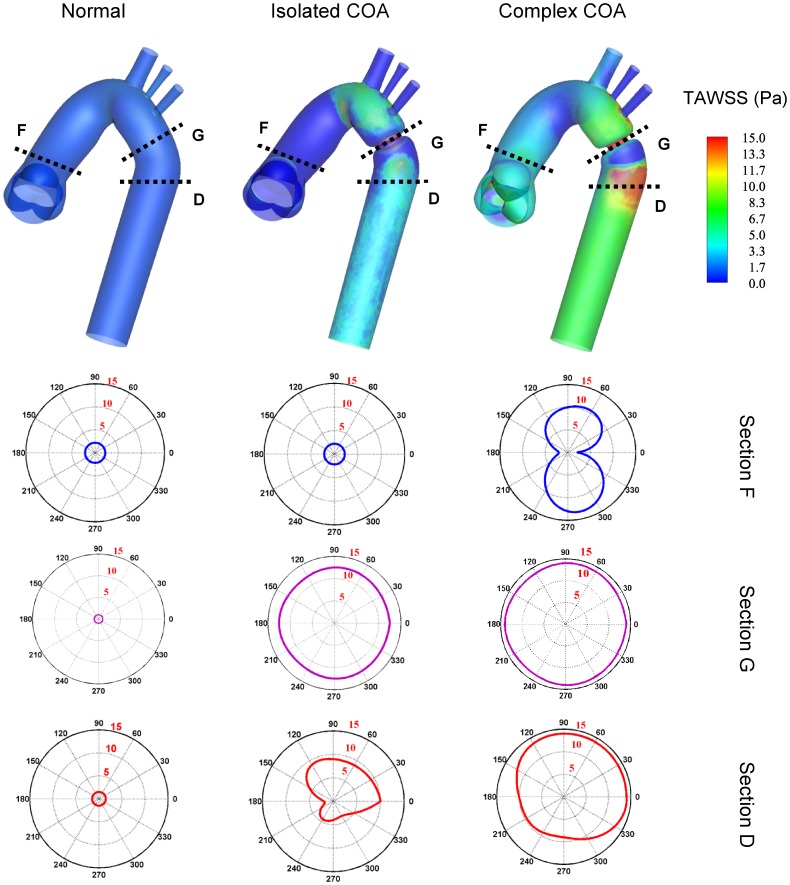
Time-averaged wall shear stress (TAWSS) contours. Downstream of the aortic valve (section F), at the isthmus zone (section G) and downstream of the COA (section D).

The hemodynamic stress that is widely accepted to act directly on the endothelial cells and shown to be responsible for many diseases and complications is the wall shear stress. It can cause degeneration of the arterial vessel, atherosclerosis, and aneurysm initiation [13], [44], [45], [46], [47]. To study the effect of hemodynamic forces on the vessel walls of an isolated COA, LaDisa et al. (2011a and 2011b) investigated the WSS and showed that a greater percentage of vessels was exposed to subnormal TAWSS or elevated OSI for COA patients [48], [49]. The total shear stress exerted on the aorta wall was evaluated using time-averaged wall shear stress (TAWSS) (Fig. 11). The specific values corresponding to a cross section located downstream of the aortic valve and along the descending aorta are also displayed. The normal case induces low values of TAWSS with a maximum of 2 Pa just downstream of the aortic valve. The presence of an isolated COA greatly increases TAWSS reaching 12.5 Pa at the neck of the COA. Elevated TAWSS could also be noticed just downstream of the COA. Now, while keeping the same COA, the presence of a BAV, leading to a complex COA, increases the TAWSS even further reaching values around 14 Pa. Very high values of TAWSS also exist downstream of the COA. This elevated and non-uniform distribution of the TAWSS downstream of the COA might lead to cellular-level changes in the vasculature [50], contributing to an increase in the stress on aortic wall and eventually aortic dissection, often reported at this location in cases of COA with BAV [5].

**Figure 12:**
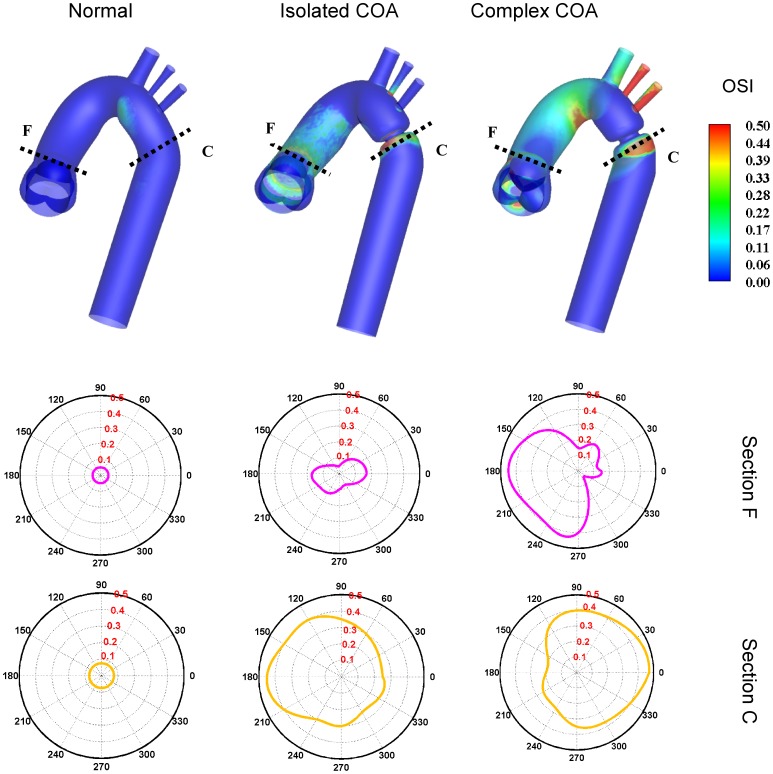
Oscillatory shear index (OSI) distribution. Downstream of the aortic valve (section F) and downstream of the COA (section C).

Figure 12 shows the oscillatory shear index (OSI) distribution which has a range between 0 and 0.5, where 0.5 indicates a purely oscillatory flow. The numerical results suggest that high OSI values of up to 0.50 can be seen downstream of the COA. Areas of high OSI (Fig. 12) lie within the areas of low TAWSS (Fig. 11) indicating flow reversal or varying flow direction (Figures 4, 5 and 7) which are considered to be more susceptible to atherosclerotic plaque formation [44], [45]. Furthermore, it should be noted that complex COA intensifies OSI magnitude with a larger affected area downstream of the COA compared to isolated COA.

**Figure 13:**
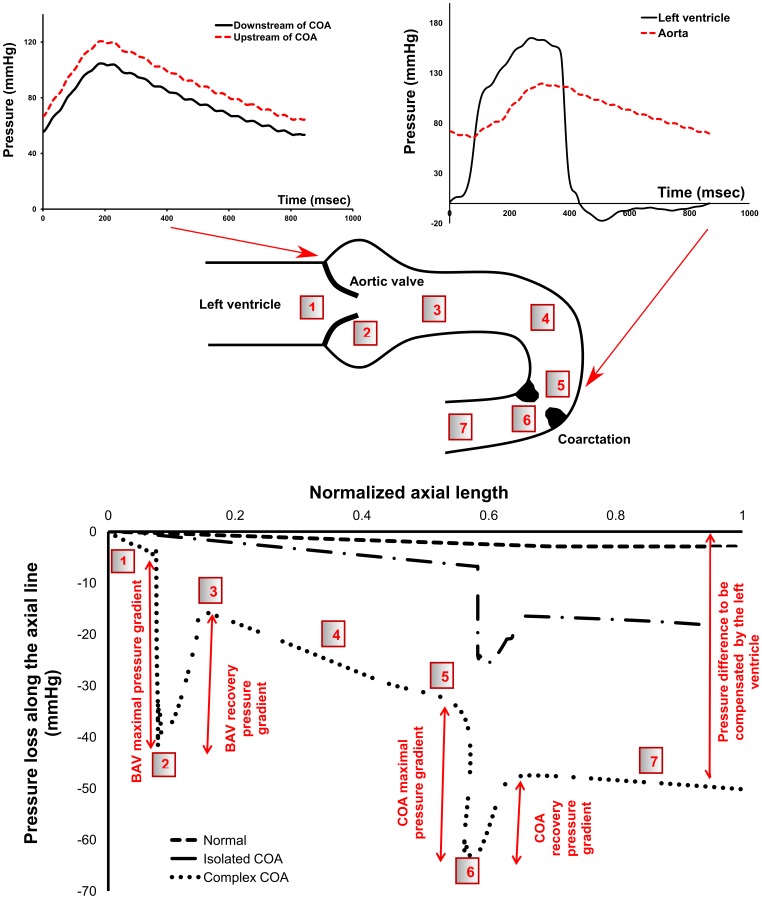
Pressure in complex COA. First row: unfiltered pressure wave forms obtained from *in vitro* model in complex COA, second row: sketch of the pressure variation along the aorta, third row: pressure loss variation at the peak of the systole along the aorta obtained numerically.

Except shear stress, the arterial blood vessel is subjected to another major hemodynamic force, pressure. Pressure loss along the central line is displayed in Fig. 13. In the normal case, the pressure drop is small and almost linear downstream of the aortic valve throughout the aorta. In the isolated COA, at the neck of the COA, the acceleration of the flow reduces the local pressure significantly. Then, flow in the descending aorta, beyond the COA, encounters an expansion causing the pressure to recover to some extent. While keeping the same COA, the presence of a BAV, leading to a complex COA, induces an increase in the maximal pressure drop at the neck of the COA. This is very important since wall compression and collapse are caused by the negative flow pressure [51] which is caused by high flow velocity due to the presence of the COA. Because of higher pressure drops in the complex COA, collapse of the vessel is more likely to happen compared to isolated COA [52]. In addition, the final recovered pressure due to the flow expansion in the descending aorta is significantly lower in the complex COA compared to isolated COA, leading to a significant pressure drop at the outlet. This pressure drop has to be compensated by the left ventricle and can lead to heart failure [2], [9]. When COA coexists with BAV, the burden on the left ventricle significantly increases compared to isolated COA.

## Discussion

Several clinical implications can be deduced from the results obtained from this study: (1) the presence of a BAV in the complex COA case significantly increases blood flow velocity downstream of a COA comparing to the isolated COA. In this study for a same COA severity of 75%, the peak velocity downstream of the COA increased from 1.9 m/s for isolated COA to 3.0 m/s for complex COA. As a result, several Doppler echocardiographic parameters based on the determination of maximal velocity downstream of the COA (maximal post-COA velocity, maximal and mean trans-COA Doppler pressure gradient) will overestimate the severity of the obstruction in cases where BAV is present. Parameters independent of post-COA velocity, like COA Doppler velocity index or COA effective orifice area, recently suggested by Keshavarz-Motamed et al. (2012), will provide a more accurate prediction of the severity of the COA independently from the flow rate and/or aortic valve conditions (bicuspid aortic valve and tricuspid aortic stenosis) [36]; (2) The results of the current study demonstrate that BAV interacts with COA fluid dynamics, amplifying irregular flow patterns and consequently TAWSS and OSI especially downstream of COA. This means that BAV may contribute to speed up the progression of diseases in this region, such as atherosclerosis, and may lead to major aortic wall complications such as aortic aneurysm [24], [50], [53], [54], rupture [55], [56] and dissection. Furthermore, when the COA coexists with the BAV, leading to complex COA, the high pressure loss downstream of the COA can augment the flow resistance and lead to collapse the wall [51], [52]. In addition, the burden on the left ventricle significantly increases which can lead to heart failure [2], [9]. The results suggest that not only the severity of the COA should be considered to evaluate the mentioned risks but also the presence and the severity of the BAV; (3) The findings of this study can help hypothesizing in part the mechanisms behind the aneurysm development in the aorta before or after a surgical repair of the COA. The specific alterations in the flow field and induced complications resulting from the sole presence of a BAV can be found in the literature [57]–[64]. The development of an aneurysm involves the interplay between the mechanical forces applied to the artery and the biological processes occurring at the cellular level [65]. When the COA and the BAV coexist, prior to surgery, TAWSS is significantly high upstream and downstream of the obstruction. Furthermore, pressure wave reflection at the obstruction site leads to an elevated systolic pressure in the ascending aorta [10], [66]. Given that patients with BAV have thinner elastic lamellae of the aortic media and a greater distance between the elastic lamellae, when compared to patients with normal tricuspid aortic valve^5^, then this combination of degenerated aortic wall and elevated systolic aortic pressure might explain the preponderance of ascending aorta aneurysm prior to the surgery. Post-surgery, the pressure in the ascending aorta is reduced since the main site of pressure wave reflection has been removed. However, in the descending aorta removal of the obstruction results in a local increase in blood pressure since: 1) the flow rate in the descending aorta will increase [36], 2) the main site of pressure loss (the COA) no longer exists. Therefore, the degenerated descending aorta wall is now subjected to an elevated pressure regime, compared to the pre-surgical condition. This might explain why aneurysm at the site of the obstruction is more common post-surgery (17 local type aneurysm vs. 8 ascending aorta aneurysm post-surgery in the study of von Kodolitch et al. (2002) [8].

## Limitations

One limitation that can be associated with our simulations is modeling of the aortic valve leaflets to be rigidly open throughout the systolic phase. However, the input velocity waveform imposed at the left ventricle outflow tract upstream of the valve varies with time and handles the variations in the flow rate crossing the aortic valve. Indeed, the main focus of this work is on the systolic phase while the aortic valve is completely open. The good agreement between numerical simulations and MRI velocity measurements, which includes moving valve leaflets, shows that this limitation does not significantly modify the conclusions of this study. Interestingly, only a limited number of interesting studies have been dedicated to quantitative study of the COA [48], [49], [67], [68]. However, in the previously reported COA studies, the aortic valve was not considered [48], [49], [67], [68]. Therefore, this work has the significant advantage of considering aortic valve geometry (tricuspid and bicuspid), while limiting the computational cost, compared to previous studies dedicated to the COA. Future numerical studies will consider the interaction between the fluid and structure and investigate the effect of dynamical opening and closing of the aortic valve leaflets on the vortex dynamics in the aorta, mainly with BAV pathology [57], [58], [59], [60], [61], [62], [63], [64], [69]. One of the most frequent complications is aorta complications (ascending and descending aortic aneurysms) in patients with the COA and the BAV. Future studies will be dedicated to the study of the effect of the BAV and aorta complications on COA fluid dynamics. Furthermore, if more details regarding transitional phenomena are of interest or if the target is to resolve the turbulence characteristics, then more computationally intensive large eddy or direct numerical simulations of turbulence are required.

## Conclusions

In this study, joint experimental (MRI) and numerical investigations were performed in different models of the aorta: normal aorta and COA (75% by area), with different aortic valve conditions: tricuspid and bicuspid and under pulsatile mean flow rate of 5 L/min. The results show that the coexistence of COA and BAV significantly alters blood flow in the aorta. Higher eccentric jet which consequently generates high pressure loss, significant reverse flow along the aorta wall, and stronger secondary flow patterns are generated downstream of the COA. As a consequence, elevated time-averaged wall shear stress and the oscillatory shear index distribution exist specifically downstream of the COA, demonstrating the interaction of BAV with COA. This can partially explain the complications associated with COA in the presence of BAV and the consequence adverse outcome post-surgery.
